# Fasting Interventions for Stress, Anxiety and Depressive Symptoms: A Systematic Review and Meta-Analysis

**DOI:** 10.3390/nu13113947

**Published:** 2021-11-05

**Authors:** Elisa Berthelot, Damien Etchecopar-Etchart, Dimitri Thellier, Christophe Lancon, Laurent Boyer, Guillaume Fond

**Affiliations:** 1 Assistance Publique-Hôpitaux de Marseille, Aix-Marseille University, Faculté de Médecine—Secteur Timone, EA 3279: CEReSS—Centre d’Etude et de Recherche sur les Services de Santé et la Qualité de vie, 27 Boulevard Jean Moulin, 13005 Marseille, France; elisa.berthelot@ap-hm.fr (E.B.); damien.etche@gmail.com (D.E.-E.); christophe.lancon@ap-hm.fr (C.L.); laurent.boyer@ap-hm.fr (L.B.); 2 Fondation Fonda Mental, 94000 Créteil, France; 3 Institut de Neuro-Épidémiologie Tropicale, Université de Limoges, 27 Boulevard Jean Moulin, 13005 Marseille, France; dthellier@icloud.com

**Keywords:** public health, mental health, fasting, antidepressant, depression, anxiety, schizophrenia, physical health, obesity

## Abstract

Background. Fasting interventions have shown effectiveness in alleviating stress, anxiety and depressive symptoms. However, no quantitative analysis has been carried out thus far. The objective was to determine the effectiveness of fasting interventions on stress, anxiety and depression and if these interventions were associated with increased or decreased fatigue/energy. Methods. Overall, 11 studies and 1436 participants were included in the quantitative analyses. Results. After limiting analyses to randomized controlled trials with low risk of bias, we found that fasting groups had lower anxiety (b = −0.508, *p* = 0.038), depression levels (b= −0.281, *p* = 0.012) and body mass index compared to controls without increased fatigue. There was no publication bias and no heterogeneity for these results. These interventions were safe, even in patients with type 2 diabetes. Conclusions. These results should be taken with a caveat. These results are preliminary and encouraging and fasting appears to be a safe intervention. Data are not sufficient to recommend one fasting intervention more than the others. No study was carried out in psychiatric populations and further trials should be carried out in these populations that may be good candidates for fasting interventions.

## 1. Introduction

Depressive and anxiety disorders are leading worldwide causes of disability and loss of quality-adjusted life year in people aged < 40 years [1]. Antidepressants are the gold standard treatments for these disorders but are effective in only approximately half of the patients and induce frequent side effects. Identifying new pathophysiological pathways to develop personalized treatments for these disorders and improve the benefit/risk ratio is a major challenge of current research. Among these new pathways, the gut–brain axis has generated a lot of interest with the recent discoveries concerning the microbiota and its role in anxiety and depression [2,3]. The field of psychonutrition has developed in parallel with the discovery of the protective role of a healthy/anti-inflammatory diet on depression onset [4,5] and the effectiveness, among other nutrients, of omega 3 fatty acids in the treatment of anxiety and depression [6]. 

In the 1990s and 2000s, some trials explored the effect of therapeutic fasting (or very low-caloric fasting) on depression and anxiety with inconsistent results and without non-fasting group control [7,8,9]. Fasting interventions are becoming in parallel more and more popular in the general population. Individuals experiencing these fasts mostly report a subjective psychological improvement.

Intermittent fasting is defined by reducing the daily duration of diet intake. Intermittent fasting can take different forms, from fasting one or two days a week to fasting 12 to 18 h a day. The potential effectiveness of intermittent fasting on mood has raised growing interest. Overweight/obesity is associated with increased depression and fasting may be effective in improving depressive symptoms by favoring weight loss [10,11,12,13]. In addition to weight loss, rodent and human studies have shown that daily intermittent fasting may switch glucose metabolism to ketone metabolism, inducing anti-inflammatory, anti-oxidative and stress resistance effects [14]. Fasting may improve microbiota disturbances and intestinal inflammation through decreased inflammatory foods intake and decreased blood flow dedicated to digestion [15]. The safety and acceptability of intermittent fasting may be limitations to the development of fasting interventions. Among them, it is unclear if fasting interventions may decrease energy/increase fatigue. This question is of importance as fatigue is a common depressive symptom [16]. Another remaining question is that intermittent fasting is often combined with caloric restriction, and there is a debate to know which is the true effective intervention to improve anxiety and depressive symptoms. To address this question, a randomized controlled trial has been carried out comparing caloric restriction with or without 14 h of restricted feeding in type 2 diabetes patients [17]. The authors concluded that both regimens were associated with improved depression, suggesting that caloric restriction should also be studied among fasting interventions. 

The primary objective of this systematic review and meta-analysis was to determine the effectiveness of fasting interventions on stress, anxiety and depression. The secondary objective was to confirm that these interventions were also effective in reducing body mass index and if these interventions were associated with increased or decreased fatigue/energy. 

## 2. Materials and Methods

### 2.1. Literature Search Strategy 

This meta-analysis was conducted in accordance with the Preferred Reporting Items for Systematic reviews and Meta-Analysis guidelines. Systematic bibliographic searches were carried out according to the Cochrane methodology. This project was registered in PROSPERO (reference number CRD42020197359) (https://www.crd.york.ac.uk/prospero/ accessed on 18 August 2020). The search paradigm was based on the PubMed interface (Medline database) and adapted for 2 databases: ScienceDirect and Google Scholar. There were no restrictions for languages and dates. The search paradigm was based on the following combination of MeSH terms “fasting” AND each MeSH terms: “anxiety” OR “stress” OR “mood disorder” OR “depression” OR “depressive”. In the case of missing data, authors were contacted by email if possible. The reference lists and bibliographies of relevant reviews and articles retrieved from the database searches were manually searched for additional eligible articles. The last search was carried out on 30 August 2021.

### 2.2. Eligibility 

The inclusion criteria were: (1) any language and date of publication; (2) original research papers; (3) fasting intervention; (4) evaluation of stress and/or anxiety symptoms and/or depression after one fasting intervention with a validated scale; (5) observational studies or controlled trials. The exclusion criterion was diet interventions not directly targeting fasting or caloric restriction. The titles and abstracts were screened by 2 researchers (E.B. and G.F). The full texts of the manuscripts were then reviewed to determine whether a study would be included (E.B. and G.F). In the case of non-consensus, a third author (L.B.) had the final decision for inclusion. 

### 2.3. Data Extraction 

Two researchers (E.B. and G.F.) extracted data from the included studies in a systematic manner using a predesigned extraction form. Each discrepancy in data extraction was examined by three authors (E.B, D.E.E and G.F) to reach consensus.

The variables were extracted as follows: study ID and design, author, year, type of study, sample size, fasting intervention description, fasting duration, religious fasting (y/*n*), fasting intervention including caloric restriction (y/*n*), socio-demographic data (country, mean age, percentage of men, study including clinical population vs. healthy volunteers, baseline and post-intervention body mass index (mean/standard deviation (SD)), baseline and post-intervention stress, anxiety and depressive symptoms scores in fasting and control groups (mean (SD)), delay between the end of fasting intervention and first evaluation (weeks), and number and type of potential adverse event. 

### 2.4. Study Quality

The study quality was assessed by DEE and GF with the study quality assessment tool for observational cohort or cross-sectional studies for Ramadan studies and for quality assessment tools for controlled studies for fasting intervention controlled trials [18]. In cases of non-consensus, a third author (L.B.) made the final decision for study quality.

### 2.5. Statistical Analyses 

As Ramadan studies were all observational except one [19], we calculated a pre/post Ramadan effect. For controlled trials, we calculated mean standardized estimate between groups receiving fasting intervention vs. controls at the endpoint following the end of fasting intervention (i.e., differential changes from baseline to post-fast in the fasting versus control groups). Heterogeneity between studies was measured by Cochrane’s Q test. Publication bias was assessed using Egger’s test funnel plot. We used comprehensive meta-analysis software (v3.0), Biostat, Englewood, NJ 07631, USA.

### 2.6. Role of the Funding Source 

This work received no funding. No drug manufacturing company was involved in the study design, the data collection, the data analysis, the data interpretation, the writing of the report, or the decision to submit the report for publication.

## 3. Results

### 3.1. Characteristics of Studies

**Figure 1:**
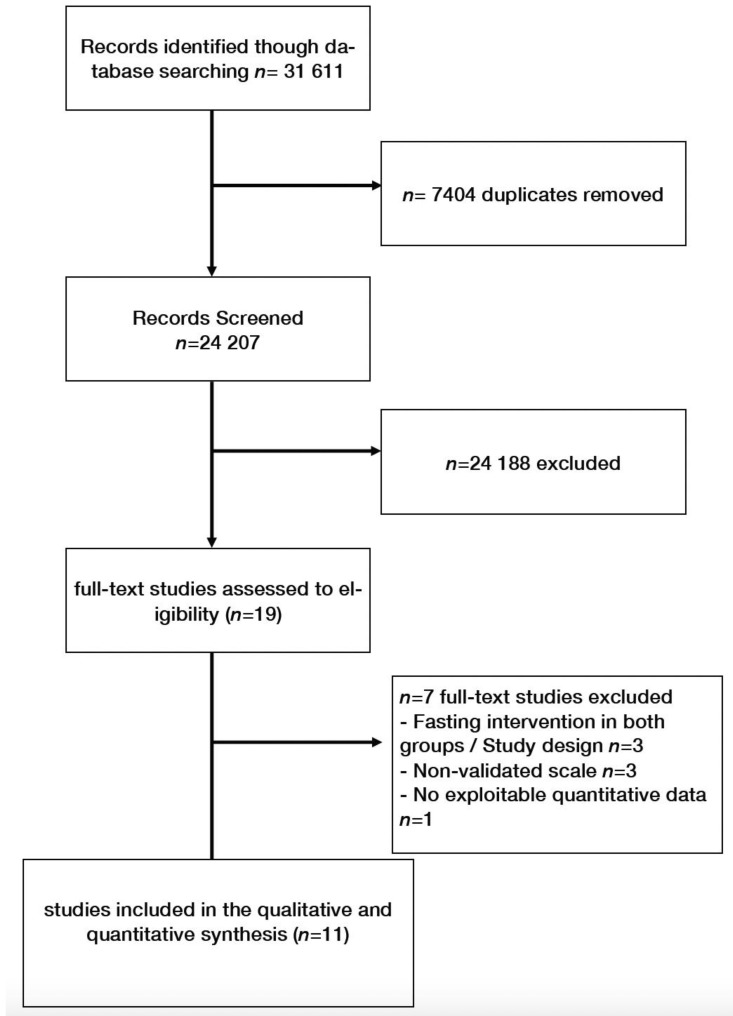
Flow chart.

Overall, 11 studies were included [17,19,20,21,22,23,24,25,26,27,28] (flow chart, Figure 1). Among the 1436 participants, 1009 subjects experienced Ramadan fasting, 239 received other fasting interventions (2 days/week (*N* = 28) or one day/week fasting (*N* = 22), 14 h restricted fasting with caloric restriction (*N* = 27) or caloric restriction without intermittent fasting (25% caloric restriction or 800 cal/day) (*N* = 162)). One study was included in both Ramadan and fasting controlled studies [19]. Three very low-calorie diet interventions could not be included because both groups received fasting interventions [7,8,9].

### 3.2. Ramadan Studies

**Figure 2:**
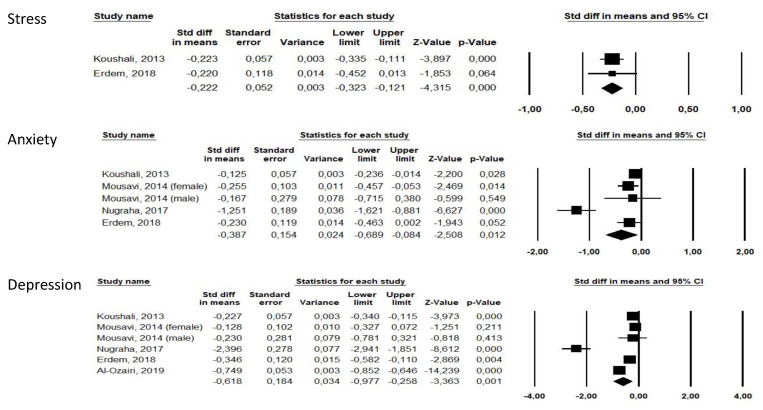
Forests plots of Ramadan studies for stress, anxiety and depression.

Ramadan study (Figure 2) characteristics are presented in Table 1 and study quality in Supplementary Table S1. Overall, five studies (1009 participants) [19,22,23,26,28] were included in the Ramadan studies. Two studies were carried out in Iran [22,23], one in Germany [19], one in Turkey [26], and one in Kuwait [28]. One study [22] was carried out in hospital nurses, one in a type II diabetes mellitus population [28], and the others in healthy volunteers [23].

Overall, Ramadan was associated with improved stress (b = −0.222 [−0.323;−0.121], *p* < 0.0001, I2 = 0), improved anxiety (b = −0.387 [−0.689;−0.084], *p* = 0.012, I2 = 87.79) and improved depression (b = −0.618 [−0.977;−0.258], *p* = 0.001, I2 = 95.32).

Four studies were classified with moderate risk of bias [19,23,26,28] and one with high risk of bias [22] (Supplementary Table S1). Removing this study did not change our results. The moderate risk of bias was due to studies using self-reported questionnaires and participants being aware of the exposure, as for all nutritional intervention studies. 

Funnel plots for Ramadan studies are presented in Supplementary Figure S1. We found no publication bias (Egger’s tests > 0.05 for anxiety and depression).

The observational Ramadan studies did not report fasting’s adverse events [22,23,26,28]. Fatigue was reported only in the controlled study [19]. Ramadan was associated with increased fatigue during the first week but decreased fatigue during week 2 to 4 and decreased sleepiness during the whole of Ramadan. No study reported dropout due to inability to follow Ramadan.

### 3.3. Fasting Controlled Trials 

**Figure 3:**
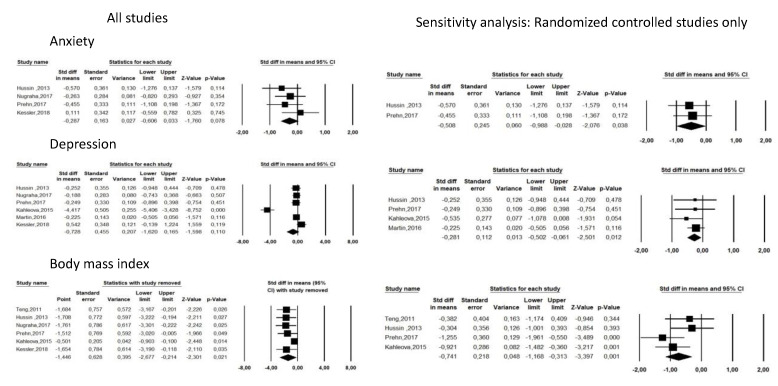
Forest plots of fasting intervention controlled studies for anxiety, depression and body mass index.

Fasting controlled trials (Figure 3) characteristics are presented in Table 1 and study quality in Supplementary Table S2. Seven studies (452 participants, 264 receiving fasting intervention, 188 being controls) were included (five randomized controlled trials [17,20,21,24,25] and two controlled trials [19,27]). The two studies carried out in Malaysia assessed the effectiveness of a 12-week, 300–500 kCal daily caloric restriction associated with 2 days a week of Sunnah Muslim fasting [20,21]. One study carried out in the Czech Republic studied the effects of 12 weeks of caloric restriction with or without 14 h/day intermittent fasting in a diabetes population [17]. One US study measured the effects of 104 weeks of 25% caloric restriction [24]. Among the three studies carried out in Germany, one studied the effects of Ramadan [19], one the effects of 12 weeks of an 800 Cal/day low calorie diet [25] and one the effects of 8 weeks of one day per week fasting, totaling 24 h a week [27]. 

Overall, the fasting groups were not found to have lower anxiety or depression levels compared to control groups at the end of fasting (*p* > 0.05, Figure 3), but they were found to have lower body mass index (b = −1.446[−2.677;−0.274], *p* = 0.021). 

After removing the two non-randomized controlled trials [19,27], fasting groups were found to have lower anxiety and depression levels (respectively, b = −0.508[−0.988;−0.028], *p* = 0.038, I2 = 0 and b = −0.281[−0.502;−0.061], *p* = 0.012, I2 = 0).

Four randomized controlled trials were evaluated to have low risk of bias [17,20,21,24], one with intermediate risk of bias [25] and the two controlled trials were evaluated to have a high risk of bias [19,27].

There was no publication bias for anxiety and depression (Egger’s tests > 0.05) but a publication bias for body mass index (Egger’s test = 0.01). Funnel plots are presented in Supplementary Figure S2.

Fatigue was measured in four studies [19,21,24,27]. The fasting groups were not found to have lower or increased fatigue levels at the end of fasting compared to control groups (*p* > 0.05, data not shown). Limiting the analysis to randomized controlled trials [21,24] did not change our results.

Overall, 42 (15.4%) dropouts were reported in fasting groups and 20 (10.2%) in control groups (*p* > 0.05). Among dropouts of fasting interventions, two diabetic patients reported a lack of motivation for 2 meals a day [17], two participants were unable to follow the two days/week fasting combined with caloric restriction for 12 weeks [20] and three participants were removed for safety reasons for 104 weeks of 25% caloric restriction [24]. The other dropouts were for reasons not related to fasting or for unknown reasons. 

Only one study reported detailed adverse events for 1 day/week fasting [27]. These adverse events were: headache, migraine, nausea, ravenousness, circulatory disturbance, hunger, general feeling of weakness, tiredness, stomach ache, meteorism, heartburn, and cold sensations in the body.

## 4. Discussion

In our meta-analysis including 11 studies and 1436 participants, we found that post-Ramadan scores for stress, anxiety and depression were lower compared to those before Ramadan. In fasting controlled trials, we found no significant effect of fasting on anxiety and depression when analyzing all studies. However, we found that fasting groups had lower anxiety and depression levels compared to control groups when limiting the analyses to randomized controlled trials. Fasting was associated with decreased body mass index in all studies without increased fatigue in fasting groups compared to controls. Adverse events were only reported for 1 day/week fasting. 

First, we found a positive effect of Ramadan on stress, anxiety and depression. Ramadan is a religious fasting, i.e., including a spiritual and social dimension that may be missing in other forms of fasting. One may hypothesize that depression improvement may not be only due to fasting but also to other lifestyle modifications. For example, Ramadan fasting includes tobacco abstinence, and tobacco abstinence has been associated with improved depressive symptoms [29]. As Ramadan is a dry fasting between sunrise and sunset, the Ramadan fasters may wake up earlier in the morning to feed before sunrise and may therefore reduce their sleep duration. Sleep reduction has been associated with depression improvement [30] and may also play a role in the observed results. Despite its significance, we found heterogeneous results across Ramadan studies for anxiety and depression. We have identified the following factors that may explain this heterogeneity: country/cultural context, clinical (diabetes) vs. non clinical populations, various delay between end of Ramadan and first endpoint evaluation (from 0 to 6 weeks), various scales to assess stress, anxiety and depressive symptoms, various ages and sex ratios. These variables could not be tested due to the small number of studies. Other uncaptured data, such as socioeconomic environment, addictive behaviors, sleep, diet, physical activity and physical comorbidities, including overweight/obesity, may also contribute to heterogeneity. For example, the German study included only healthy male students [19] and the effect of Ramadan on anxiety was much higher in this study compared to the others. However, the results were still significant after removing this study. It should be underlined that all scales to evaluate stress, anxiety or depression were self-reported, and that clinician-rated scales could be useful to confirm these results. 

The second major finding is the result of fasting controlled trials. Our overall results were not significant (with a trend toward significance for anxiety, *p* = 0.07). However, after removing two studies with a high risk of bias, the results became significant with low heterogeneity. These results are encouraging to pursue research on fasting intervention effects on mental health, especially in psychiatric samples that have been untested thus far. It should be underlined that the mean stress/anxiety/depression scores were mostly under the pathological rank at baseline, suggesting that fasting interventions are effective for moving from a “healthy” to “even healthier” mood. Despite this fact, the effect sizes indicated a mild effect for depression and moderate effect for anxiety when limited to randomized controlled trials. As most interventions are most effective in patients with more severe baseline symptoms, we believe that fasting interventions should therefore be effective in psychiatric samples. Moreover, we found that patients receiving these interventions benefited from body mass index reduction. Obesity is common in patients with major depression and may influence psychiatric trajectory [31,32]. Intentional weight loss improves the symptoms of depression [10]. However, no study explored if fasting interventions were more effective in overweight participants and it remains to be determined if body mass index is correlated with depressive symptoms. It remains also to be determined if overeating is conversely associated with impaired mood and the direction of the causal relationship [33]. Animal studies also suggest that fasting improves oxidative stress [34]. Further studies should explore if the improvement of oxidative stress parameters would be associated with improved anxiety/depression in humans.

Adverse events were poorly reported. The only study reporting adverse events was those exploring the effects of 24 h/week fasting. Daily intermittent fasting may be effective in limiting these adverse events. The absence of significant dropout rate differences between fasting and control groups is encouraging for the acceptability of fasting interventions. It should be underlined that two meals/day associated with caloric restriction appeared as a safe intervention in patients with diabetes. No hypoglycemia was reported in this study. Metabolic disorders are frequent in psychiatric populations and should therefore not be a limitation to test fasting interventions in psychiatry. Additional data are needed to confirm these preliminary results.

Strengths. We used the most recent meta-analysis standards to carry out the present meta-analysis. A comprehensive search following PRISMA criteria has been carried out, and we used leave-1-out analyses and quality evaluations to determine the risk of bias. The present work therefore adds important knowledge in the field.

Limitations. Our results must be interpreted with caution. Only 11 studies with relatively small sample sizes were included. Four Ramadan studies were observational and two fasting controlled trials were not randomized. The small number of studies did not enable us to carry out sensitivity analyses. An important limitation and direction for future research is that our results were insufficient to determine if caloric restriction is the true effective intervention to improve anxiety or depression, or if intermittent fasting (time-restricted feeding) may have a specific effect. Lastly, although visual inspection of funnel plots did not suggest publication bias, definitive confidence in excluding publication bias was limited by the small number of studies included in our funnel plots. Very low-calorie interventions could not be included in the quantitative analyses. However, most of them reported mood improvement after these interventions. Further trials are therefore warranted to explore their effectiveness.

## 5. Conclusions

Preliminary evidence suggests that fasting interventions may have a positive effect on anxiety, depression and body mass index reduction without increasing fatigue. It remains unknown if caloric restriction is the true effective component of fasting or if intermittent fasting may increase its effectiveness on stress, anxiety and depressive symptoms. A 12-week intermittent fasting associated with caloric restriction appears as a safe and acceptable intervention, even in patients with diabetes. Further randomized controlled trials are warranted to strengthen these results, especially in psychiatric populations that have not been tested thus far. 

## Figures and Tables

**Table 1:** Study characteristics.

Study	Country	N	N F	N C	N(%) Men	Design	Population	Fasting Intervention	Controls	Endpoint *	Scales **	Authors’ Conclusion	Adverse Events	N Dropout Fasting	N Dropout Controls
Koushali (2013) [22]	Iran	313	313	NA	177(56.5%)	OBS	Hospital nurses	Ramadan	NA	1 to 2	Anxiety: DASS21 Depression: DASS21	Depression and stress were significantly reduced (*p* < 0.05) but not anxiety.	md	NA	NA
Mousavi (2014) [23]	Iran	110	110	NA	13(11.8%)	OBS	Residents of Kermanshah city	Ramadan	NA	MD	Anxiety: GHQ subscore Depression: GHQ subscore Stress: GHQ	Significant reduction in anxiety (*p* = 0.011) but no significant reduction in depression (*p* > 0.05) after Ramadan.	md	NA	NA
Erdem (2018) [26]	Turkey	73	73	NA	63(86.3%)	OBS	Muslim healthy volunteers	Ramadan	NA	0	Stress: DASS-42 Anxiety: DASS anxiety Depression: DASS	Significant reduction in depression (*p* = 0.001), anxiety (*p* = 0.01) and stress (*p* = 0.002) scores after Ramadan.	md	NA	NA
Al-Ozairi (2019) [28]	Kuwait	463	463	NA	251(54.2%)	OBS	Type 2 diabetes Muslim patients ≥21 years	Ramadan	NA	4–6	Depression: PHQ-9	Significant reduction in depressive symptoms after Ramadan (*p* < 0.05).	md	NA	NA
Nugraha (2017) [19]	Germany	50	25	25	50(100%)	CT	Healthy male volunteers ≥ 18 years (mostly students)	Ramadan	No fasting and no other intervention	1	Anxiety: HADS Depression: BDI-II	Significant reduction in depressive symptoms after Ramadan (*p* < 0.05).	Increased fatigue during first week of Ramadan, then decreased fatigue during week 2 to 4 but decreased sleepiness during whole Ramadan.	3/28(10.7%) (2 time schedule, 1 other reason)	2/28(7.6%) (other reason)
Teng (2011) [20]	Malaysia	25	12	13	25(100%)	CT	Healthy men aged 50 to 70 years, BMI 23.0 to 29.9 kg/m^2^	Reduction in 300 to 500 kcal/day from thei habitual energy intake + two days of Muslim sunnah * fasting per week 12 weeks	No fasting and no other intervention	0	Depression: BDI-II	Non-significant reduction in depressive symptoms after fasting intervention (*p* > 0.05).	Adverse events were not reported but 2 participants were unable to follow the fasting intervention	2/14(14.2%) (unable to follow the fasting intervention)	1/14(7.1%) (personal reasons)
Hussin (2013) [21]	Malaysia	32	16	16	32(100%)	RCT	Healthy men aged 50 to 70 years, BMI 23.0 to 29.9 kg/m^2^	Reduction of 300 to 500 kcal/day from thei habitual energy intake + two days of Muslim sunnah * fasting per week 12 weeks	No fasting and no other intervention	0	Depression: BDI-II. Fatigue: POMS	Non-significant reduction in depressive symptoms after fasting intervention (*p* > 0.05).	No reported adverse events.	0(0%)	1/16(6.2%)
Kahleova (2015) [17]	Czech Rebublic	54	27	27	29(54 %)	RCT	Patient with type 2 diabetes, mean age 59.4 years, mean BMI 32.6 kg/m^2^	Time Restricted feeding (14 h fasting/day) + caloric restriction 12 weeks	6 meals/day (3 meals + 3 snacks)	0	Depression: BDI-II	Significant reduction in depression score was decreased in the fasting group (*p* < 0.05), and feelings of hunger were greater than in the control group. Quality of life increased (*p* < 0.01) comparably under both regimens.	No reported adverse events.	3/27(11.1%) (1 personal reasons, 2 lack of motivation)	4/27(14.8%) (2 personal reasons, 2 lack of motivation)
Martin(2016) [24]	USA	218	143	75	66(30%)	RCT	Healthy men aged 20 to 50 years and women aged 20 to 47 years, with a BMI between 22.0 and 28.0	25% Caloric Restriction 104 weeks	No fasting and no other intervention	0	Depression: BDI-II Fatigue: POMS	Significant improvement in the depression score (*p* < 0.05), tension (*p* < 0.01), and General health (*p* < 0.001).	No reported adverse events but 3/117(2.6%) participants of the fasting group were removed for safety reasons (not detailed).	26/143(18.2%) (8 withdrew consent, 6 moved away from study site, 6 for personal and other reasons, 3 women became pregnant, 3 withdrawn for safety)	5/75(6.7%) (3 women became pregnant, 1 withdrew consent)
Prehn (2017) [25]	Germany	37	19	18	0(0%)	RCT	Older obese women, mean age 61 years, mean BMI 35	Low calorie diet (800 kcal/J) 12 weeks	No fasting and no other intervention	0	Anxiety: STAI Depression: BDI-II	Reduction in Beck’s depression score (*p* < 0.001) and anxiety score (*p* < 0.004) in the fasting group.	No reported adverse events but 6 subjects were excluded for instruction failure without details.	5/23(21.7%) (personal reasons)	5/24(20.8%)
Kessler (2018) [27]	Germany	36	22	14	14(39)%	CT	Healthy volunteers	Fixed fasting day per week for 8 weeks, a fixed week day 8 weeks	2 groups counseling sessions for healthy diet + waiting list for fasting intervention	0	Anxiety: HADS-A Depression: HADS-D Fatigue: POMS	Significant within- group differences in the fasting group were observed after 6 months for the HADS total score, and the HADS depression and anxiety subscales, the POMS total score (including subscales for positive mood and vigor).	Adverse events: headache, migraine, nausea, ravenousness, circulatory disturbance, hunger, general feeling of weakness, tiredness, stomach ache, meteorism, heartburn, and cold sensations in the body.	*N* = 4/22 (9.1%) (2 declined to further participate, 2 lost of follow-up)	*N* = 2/14 (14.2%)

* in weeks after end of fasting intervention. ** all scales were self-reported questionnaires. BDI-II, Beck Depression Inventory. DASS, Depression Anxiety Stress Scale (DASS-42). GHQ, General Health Questionnaire. GHQ-28, General Health Questionnaire-28. HADS, Hospitalization Anxiety and Depression scale. POMS, Profile of Mood states. PHQ, Patient Health Questionnaire. STAI, State-Trait Anxiety Inventory. F, Fasting. C, Controls. MD, missing data. OBS, observational. RCT, randomized controlled trial. SD, standardized deviation. NA: not adapted.

## Supplementary Materials

The following are available online at https://www.mdpi.com/article/10.3390/nu13113947/s1, Figure S1: Funnel plots of Ramadan studies; Figure S2: Funnel plots of fasting controlled trials; Table S1: Study quality for Ramadan studies, Table S2: Study quality for fasting controlled trials.
