# Supplementary material for: Fasting Interventions for Stress, Anxiety and Depressive Symptoms: A Systematic Review and Meta-Analysis
Source: Nutrients. 2021 Nov 5;13(11):3947. doi: 10.3390/nu13113947 (PMC8624477; doi:10.3390/nu13113947)
Supplement: Supplementary file 1 [file nutrients-13-03947-s001.zip › nutrients-1426971-supplementary.pdf]

Supplementary Table S1. Quality assessment of Ramadan studies.

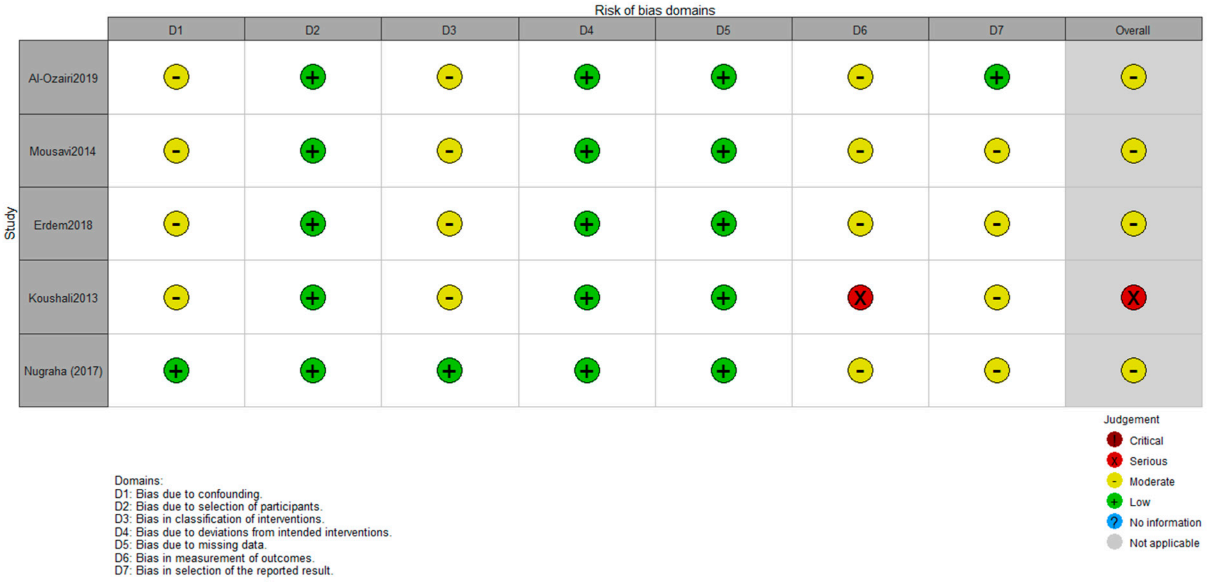

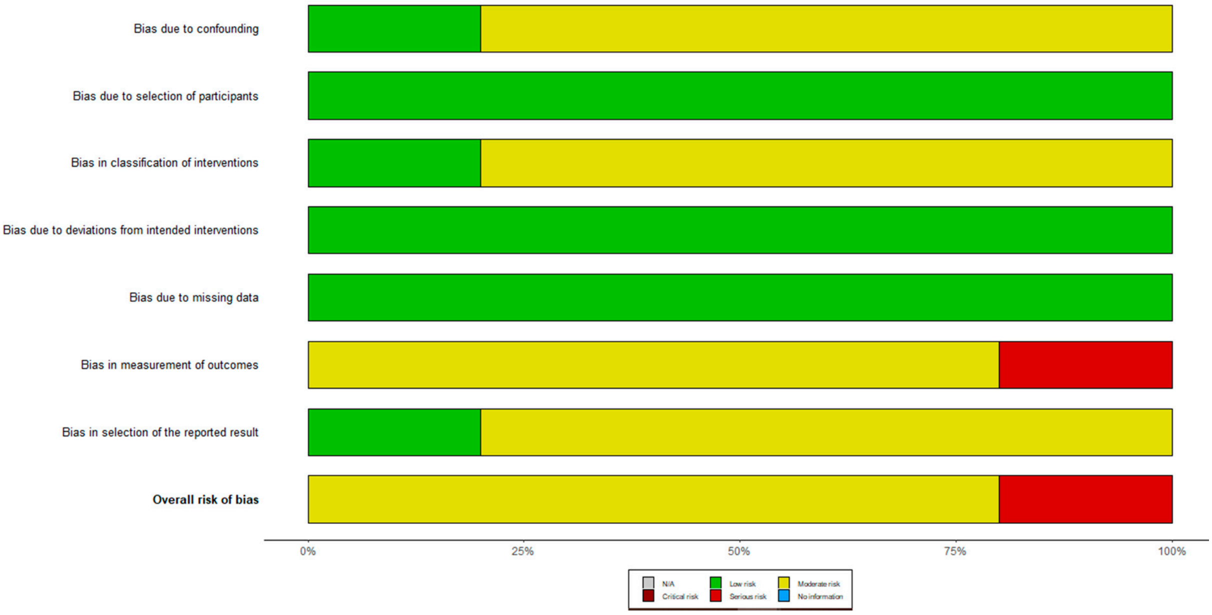

Supplementary Table S2. Quality assessment of controlled trials.

Study ID	D1	D2	D3	D4	D5	Overall
Teng2011	+	+	+	+	+	+
Hussin2013	+	+	+	+	+	+
Kahleova2015	+	+	+	+	+	+
Martin2016	+	+	+	+	+	+
Nugraha2017	-	+	+	+	+	-
Prehn2017	!	+	+	+	+	!
Kessler2018	-	+	+	+	+	-

+ Low risk

! Some concerns

- High risk

D1 Randomisation process

D2 Deviations from the intended interventions

D3 Missing outcome data

D4 Measurement of the outcome

D5 Selection of the reported result

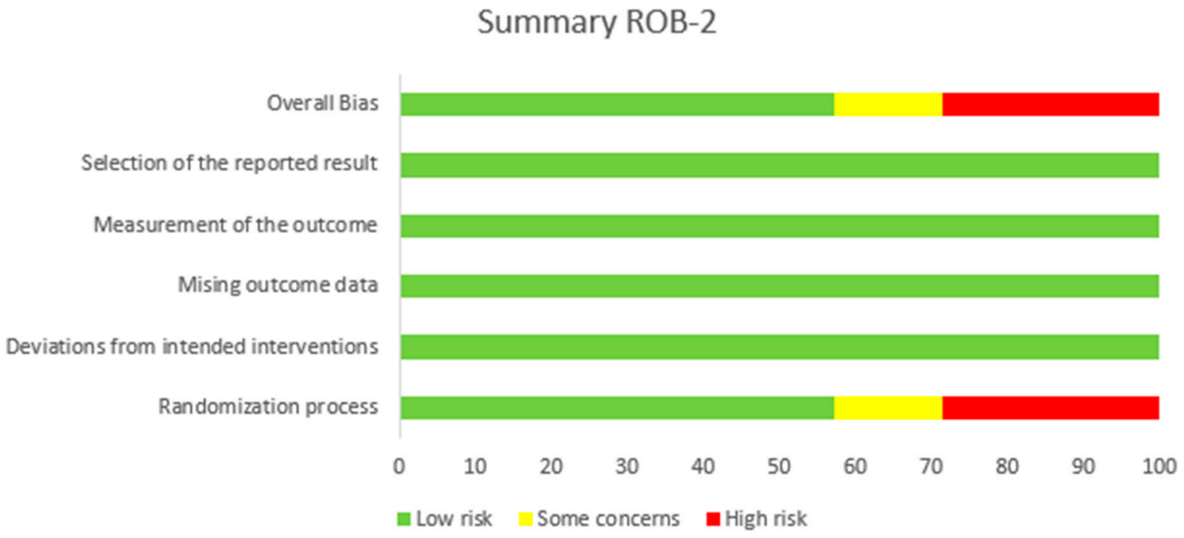

Supplementary Figure S1. Funnel plots of Ramadan studies.

Anxiety

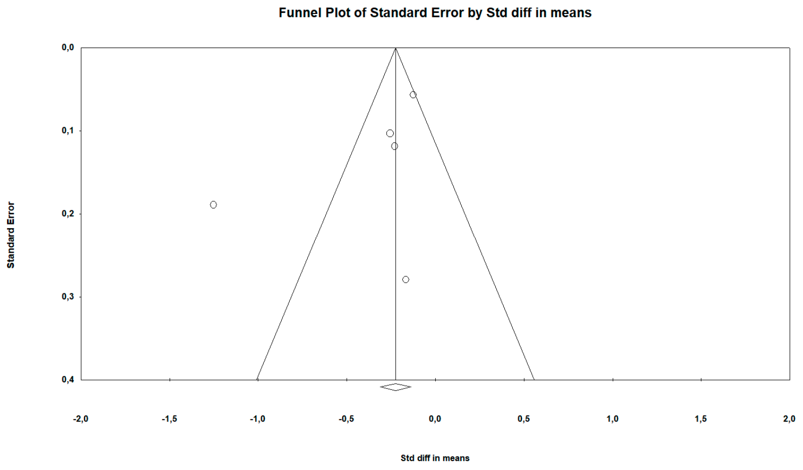

Depression

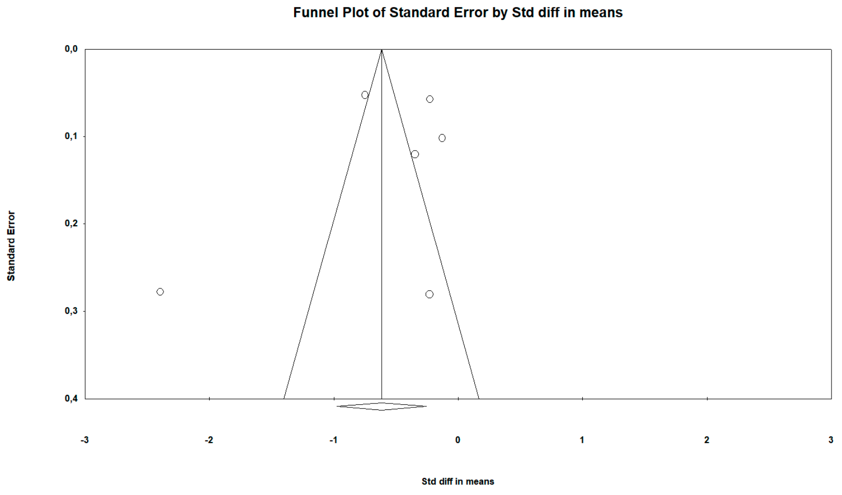

Supplementary Figure S2. Funnel plots of fasting controlled trials.

Anxiety

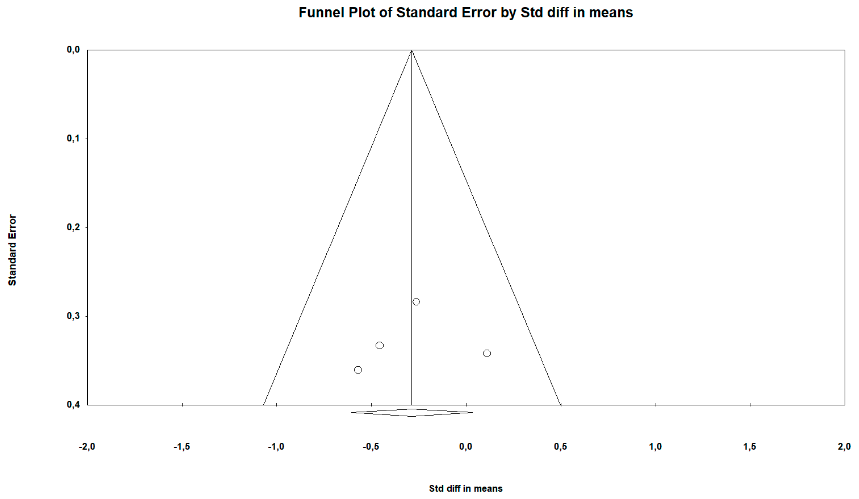

Depression

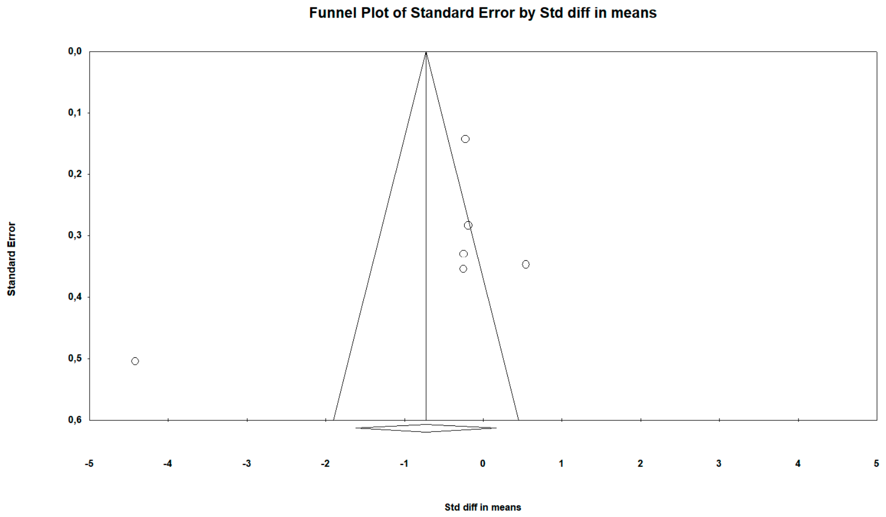

Body Mass Index

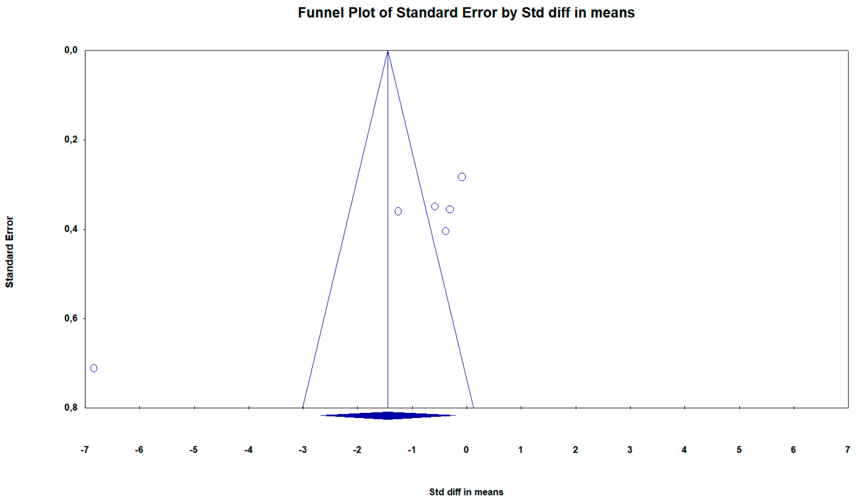

Funnel Plot of Standard Error by Std diff in means
Standard Error
0,0
0,2
0,4
0,6
0,8
-7
-6
-5
-4
-3
-2
-1
0
1
2
3
4
5
6
7
Std diff in means
